# Parallel changes in serum proteins and diffusion tensor imaging in methamphetamine-associated psychosis

**DOI:** 10.1038/srep43777

**Published:** 2017-03-02

**Authors:** Michael S. Breen, Anne Uhlmann, Sureyya Ozcan, Man Chan, Dalila Pinto, Sabine Bahn, Dan J. Stein

**Affiliations:** 1 Division of Psychiatric Genomics, Department of Psychiatry, Icahn School of Medicine at Mount Sinai, New York, New York, USA; 2 Seaver Autism Center for Research and Treatment, Icahn School of Medicine at Mount Sinai, New York, New York, USA; 3 Department of Psychiatry and Mental Health, and MRC Unit on Anxiety & Stress Disorders, University of Cape Town, South Africa; 4 Institute of Biotechnology, University of Cambridge, Cambridge, United Kingdom; 5 Institute for Genomics and Multiscale Biology, Department of Genetics and Genomic Sciences, Icahn School of Medicine at Mount Sinai, New York, New York, USA; 6 Friedman Brain Institute, Icahn School of Medicine at Mount Sinai, New York, New York, USA

## Abstract

Methamphetamine-associated psychosis (MAP) involves widespread neurocognitive and molecular deficits, however accurate diagnosis remains challenging. Integrating relationships between biological markers, brain imaging and clinical parameters may provide an improved mechanistic understanding of MAP, that could in turn drive the development of better diagnostics and treatment approaches. We applied selected reaction monitoring (SRM)-based proteomics, profiling 43 proteins in serum previously implicated in the etiology of major psychiatric disorders, and integrated these data with diffusion tensor imaging (DTI) and psychometric measurements from patients diagnosed with MAP (*N* = 12), methamphetamine dependence without psychosis (MA; *N* = 14) and healthy controls (*N* = 16). Protein analysis identified changes in APOC2 and APOH, which differed significantly in MAP compared to MA and controls. DTI analysis indicated widespread increases in mean diffusivity and radial diffusivity delineating extensive loss of white matter integrity and axon demyelination in MAP. Upon integration, several co-linear relationships between serum proteins and DTI measures reported in healthy controls were disrupted in MA and MAP groups; these involved areas of the brain critical for memory and social emotional processing. These findings suggest that serum proteomics and DTI are sensitive measures for detecting pathophysiological changes in MAP and describe a potential diagnostic fingerprint of the disorder.

Methamphetamine (METH) is a highly addictive psychostimulant severely affecting the central nervous system^1^. An estimated 0.3–1.3% of the world population abuse METH and more than 50% of all METH abusers develop psychotic symptoms^2^,^3^. The clinical presentation, course and treatment of METH-associated psychosis (MAP) overlap with that of schizophrenia (SCZ) and MAP has been put forward as an environmental and pharmacological model of SCZ^1^,^4^,^5^. Despite the clear clinical impact of METH dependence, the neural and molecular mechanisms underpinning the diverse clinical presentation of MAP remain unclear. Uncovering these pathobiological changes is critical for driving the development of improved diagnostics and treatment approaches.

Research using neuroimaging techniques has shown METH abuse is associated with abnormalities of brain function^6^, structure^7^ and receptor pharmacology^1^,^8^. However, only a few neuroimaging studies have focused on MAP. These report lower density of dopamine transporters in the nucleus accumbens^9^, grey matter reductions in the frontal cortex and limbic system^10^,^11^,^12^ and neuronal integrity deficits^13^, findings that also emerge in work on SCZ patients^14^,^15^,^16^. More recent investigations using diffusion tensor imaging (DTI), a highly sensitive technique for detecting differences in microstructure architecture, also report white matter abnormalities in the frontal brain areas related to METH dependency^17^,^18^. We recently implemented a voxel-based whole brain method for analysis of DTI data in MAP and found widespread higher levels of mean, axial and radial diffusivity together with low fractional anisotropy^19^, indicating globally impaired white matter integrity similar to that seen in SCZ. However, while these observed differences have yet to impact significantly the diagnosis or treatment of individual patients, complementary approaches to forwards these neuroimaging studies become necessary.

To this end, a growing body of research delineates changes in peripheral analyte levels that may reflect, at least in part, changes in the brain^20^,^21^,^22^,^23^,^24^,^25^,^26^,^27^,^28^. Proteomic studies of serum or plasma in SCZ^21^,^22^,^23^,^24^,^25^, bipolar disorder^26^,^27^ and major depressive disorder^28^,^29^ patients have found changes in proteins involved in inflammatory and hypothalamic-pituitary-adrenal (HPA) signaling, along with changes in hormonal, and lipid metabolism signaling. Changes in these pathways are known to correspond with alterations in brain functions such as mood, emotional responses and cognitive processes, and have also been reported in post-mortem brain tissue^21^,^22^. While no studies have directly assessed protein levels in either blood serum or post-mortem brain of MAP patients, several reports from human and murine models indicate METH use impacts protein abundance in both compartments. For example, METH has been associated with increased abundance of serum proteins, including complement factor H (CFH), transthyretin (TTR)^30^ and low-density lipoprotein (LDL) and aminotransferases^31^, as well as decreases in brain proteins, including superoxide dismutase (SOD) and glutathione peroxidase (GPx) (for review see ref. 32). A study directly measuring hormonal levels in MAP patients demonstrated increases in plasma norepinephrine^33^, suggesting a similar link may exist where alterations in inflammatory and HPA signaling mediate susceptibility to spontaneous relapse of psychotic symptoms in MAP, as reported in SCZ. We recently investigated blood RNA levels in MAP patients and described several candidate biomarkers that have also been previously found in blood and post-mortem brain tissue of psychosis and SCZ patients, including dysregulation of the ubiquitin proteasome system^34^. The convergence of these studies indicates that the brain and peripheral systems are closely connected and that similar pathways may be affected in both compartments across several disorders.

Multi-modal data integration of serum proteomics, neuroimaging and clinical measures may allow for an improved pathobiological understanding of MAP that can guide the development of improved diagnostics and treatments. Here, we applied a highly sensitive selected reaction monitoring (SRM)-based proteomics assay to assess changes in 43 proteins in serum previously implicated in the pathophysiology of major psychiatric disorders, and those related to candidate inflammatory and lipid metabolism pathways. Additionally, we collected DTI measurements from the same individuals and applied an integrated analysis across patients diagnosed with MAP, METH dependency without psychosis (MA) and healthy controls. We hypothesized that both protein expression and white matter microstructural changes in the MAP group differ significantly from MA and controls. We further explored the relationship between serum protein levels and DTI to determine how candidate proteins may relate to altered microstructural integrity in MAP.

## Methods

### Ethical approval and informed consent

The study was approved by the Faculty of Health Sciences Human Research Ethics Committee of the University of Cape Town (HREC: 340/2009) and complied with ethical guidelines established by the Declaration of Helsinki (World Medical Association, 2013). After a detailed description of the study, all participants gave written informed consent to participate.

### Participants

Three groups of age (24.45 ± 5.68) and gender (33 M/9 F) matched participants were studied based on their clinical history of METH dependence: patients with a history of METH-associated psychosis (MAP; *N* = 12), METH-dependent individuals without psychosis (MA; *N* = 14), and non-substance-abusing healthy control participants (*N* = 16). Each participant underwent two assessment sessions. The first session consisted of a detailed psychiatric interview and detailed clinical, substance and demographic variables were recorded. During the second session, approximately 1 week later, serum was collected followed by a brain scan.

Clinical assessment was undertaken using the Structured Diagnostic Interview for DSM-IV Axis I Disorders^35^ and participants completed an Eysenck personality questionnaire (EPQR-S) to assess psychoticism, extraversion and neuroticism^36^. Positive and negative symptoms within the MAP group were rated using the Positive and Negative Syndrome Scale (PANSS)^37^: PANSS positive subscale (12.5 ± 5.66), negative subscale (15.5 ± 10.05) and total score (50.58 ± 23.71). Participants were excluded from the study if they presented with: 1) additional types of substance dependence for the MA and MAP groups, and any substance dependence in the control group, with the exception of nicotine; 2) a lifetime and current diagnosis of any psychiatric disorder (other than MA dependence and MAP in the MA and MAP groups, respectively); 3) a history of psychosis prior to MA abuse; 4) a medical or neurological illness or head trauma; 5) a seropositive test for HIV; 6) MRI incompatibilities such as metal implantations or claustrophobia. The occasional use of other substances was not exclusionary. All the participants in the MAP group were on haloperidol treatment at the time of testing.

### Serum collection and preparation

Participants were required to fast overnight before specimen withdrawal and to refrain from heavy exercise, alcohol, tobacco and nicotine for 12 h. All specimens were collected between 09h00 and 11h00 using serum tubes with clot activator, and incubated at room temperature for at least 1 h until clotted. Serum aliquots were frozen at −70 °C until shipment and analysis in Cambridge, UK. Serum proteins were digested into peptides using conventional trypsin approach^38^. Briefly, proteins were reduced with 10 mM dithiothreitol (DTT) for 30 min at 60 °C and then alkylated with 10 mM iodoacetamide (IAA) for 30 min at room temperature in the dark. Overnight (17 h) tryptic digestion was performed using porcine trypsin (Sequencing Grade Modified, Promega, Wisconsin) at 37 °C. Isotopically labelled internal standard peptides were spiked into samples for prior to mass spectrometry (MS) analysis.

### Mass spectrometry analysis, quantification and pre-processing

Forty-three serum proteins implicated in the pathophysiology of major psychiatric disorders, including SCZ and psychotic disorders^25^,^39^,^40^,^41^, were monitored using Xevo TQ-S (Waters Corporation) Triple Quadrupole MS coupled with nanoAcquity UPLC system (Waters Corporation) and operated in Selective Reaction Monitoring (SRM) mode. The unique peptides representing targeted proteins and interference free transitions were selected as described previously^41^. The serum digest was first loaded into a C18 trapping column (180 um × 20 mm, 5 μm particle size) for 2 min. Peptide separations were then performed on a C18 BEH nano-column (75 um × 200 mm, 1.7 mm particle size) in a 30-min linear gradient from 3 to 40% acetonitrile containing 0.1% formic acid at the flow rate of 300 nL/min. The column temperature was 35 °C. The MS analysis was conducted in positive ion mode^42^. Each sample was analyzed in duplicate. A Skyline software package (Version 3.1.0)^43^ was used to process raw MS files and peak area values were exported for statistical analysis.

The SRM data were pre-processed using the R package MSstats^44^. The data were log_2_ transformed to stabilise variance and normalised to remove systematic bias between MS runs. The resulting profile, quality control and condition plots were carefully inspected to identify potential sources of variation for each protein, evaluate any systematic bias between MS runs and assess the variability of each condition per protein, respectively. Transitions with a between-run-interference score of less than 0.8 were excluded as described previously^45^. This score was calculated based on the correlation between mean of peptide by run and peptide transition intensity^46^. Transitions with a coefficient of variation of over 10% in over 10% of sample runs were also excluded. Singular value decomposition was implemented on all remaining transitions corresponding to a particular protein and the resulting eigengene value, equivalent to the first principal component, was used to represent the overall profiles for each protein. Data were inspected for outlying samples using unsupervised hierarchical clustering (based on Pearson coefficient and average distance metric) and principal component analysis to identify potential outliers outside two standard deviations from these averages; no outliers were present in these data. Finally, linear mixed models from the R package variancePartition^47^ were used to characterize and identify environmental drivers that may affect the observed protein abundances between groups. This approach quantifies variation in each abundance trait attributable to differences in age, gender, technical variables and importantly, environmental exposures.

### Image acquisition and analysis

Acquisition of diffusion tensor images (DTI) on a Siemens Magnetom 3 T Allegra (Erlangen, Germany) scanner has been described elsewhere^19^. In brief, a single-shot echo-planar imaging (EPI) sequence with a spatial resolution of 2 × 2 × 2 mm was used with the following parameters: TR = 9500 ms, TE = 88 ms, FOV = 240 mm, 30 isotropically distributed diffusion-weighted directions, diffusion weightings b = 0/1000 s/mm2, and transversal orientation with 70 slices and no gaps.

DTI data were analyzed using the FMRIB Software Library (FSL)^48^ and MATLAB. Fractional anisotropy (FA), mean diffusivity (MD; mean of the three eigenvalues), axial diffusivity (AD; first eigenvalue), and radial diffusivity (RD; mean of second and third eigenvalue) were calculated. Data pre-processing included eddy current and motion correction, tensor calculation, outlier rejection, and affine registration to create a mean DTI image. Tract-based spatial statistics (TBSS) was used for whole brain voxelwise analysis^49^, including the alignment into Montreal Neurological Institute-152 (MNI152) common space and the creation of a mean FA-skeleton image which individual images were projected onto. Regional summary measures of various fiber tracts were calculated by registering the FA image from the JHU ICBM-DTI-81 white-matter labels atlas^49^ to each participant. The mean of all voxels from each of the 48 regions-of-interest (ROI) from the atlas were obtained from maps of FA, MD, AD, and RD.

### Statistical analyses

All statistical analyses were conducted in the statistical package R. First, demographic, clinical and questionnaire data were analyzed with the Shapiro-Wilk test to assess normality of variables and either a one-way analysis of variance (ANOVA) or a Kruskal-Wallis ANOVA with *post hoc* Tukey correction was implemented, respectively. Categorical variables, such as gender and polysubstance use, were compared using chi-square tests. Second, protein-level quantification and testing for differential protein abundance between groups was carried out using mixed model ANOVAs, as implemented in the R package multcomp^50^. The covariates age, gender and polysubstance use were included in the models to adjust for their potential confounding influence on protein abundance between-group main effects. Pairwise *post hoc* Tukey corrections were applied to determine which comparisons were statistically different, and adjusted *P*-values < 0.05 were designated as significant. Third, in a similar fashion, regional mean diffusion parameters were compared between groups while correcting models for the aforementioned covariates and correcting *P*-values for multiple comparisons. Finally, we explored correlations of protein abundance with psychometric measurements, using Spearman correlation, and DTI measurements, using Pearson correlation, both across and within groups and *P*-values were adjusted by a conservative Bonferroni multiple test correction (0.05/3 psychometric data = *P* < 0.01; 0.05/48 ROI = *P* < 0.001). Significant correlations were inspected for outlying data-points driving the observed associations and removed accordingly.

### Data availability

Raw peptide and normalized protein datasets can be found in Supplementary Tables 1–2.

## Results

We conducted a preliminary study and integrated highly sensitive SRM-based proteomics, profiling 43 proteins in the serum with psychometric measurements and DTI from a primary cohort of 12 MAP, 14 MA, and 16 healthy controls (Table 1). We explored the hypothesis that serum proteins previously implicated in the pathophysiology of major psychiatric disorders, and those related to candidate inflammatory and metabolic pathways, mediate susceptibility for psychosis with METH abuse, and that these differences occur in parallel with white matter microstructural alterations in MAP. We further studied the relationship between serum protein levels and DTI to determine how serum protein abundance may relate to altered microstructural integrity in MAP.

### Serum proteomics

**Figure 1:**
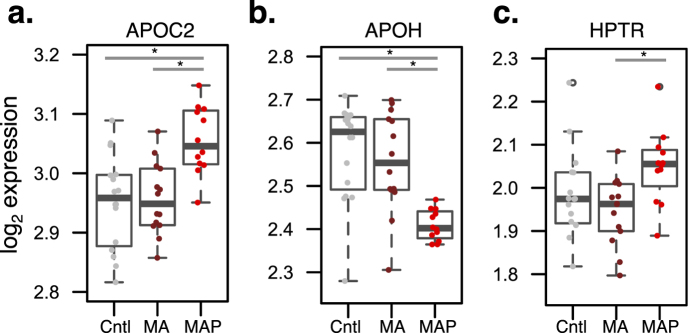
Differential protein expression analysis. The MAP group displayed differences from MA and healthy control (Cntl) groups for apolipoproteins (**a**) APOC2 and (**b**) APOH. (**c**) The MAP group also displayed differences in HPTR protein abundance from MA but not healthy controls. Mixed model analysis of variance compared protein levels between groups and controlled for age, gender and polysubstance use (nicotine, alcohol, cannabis, mandrax). Resulting *P*-values were *post hoc* Tukey corrected, and adjusted *P*-values < 0.05 were considered significant (*).

SRM-based proteomics measured 43 proteins in the serum across all 42 participants enrolled in this study (Supplementary Table 1). Following normalization and quality control of protein expression (Supplementary Figure 1), we considered whether age, gender and polysubstance use measures (nicotine, cannabis, alcohol, mandrax) may be driving protein abundance variation and sought to identify proteins that deviate from the general trend (Supplementary Figure 1D). Collectively, the median variance explained by these measures was considerably low and unable to explain the observed protein level variation. Subsequently, we assessed whether serum protein abundance differed between groups while adjusting for the above-mentioned covariates and correcting *P*-values for multiple comparisons. MAP subjects displayed significant up-regulation of apolipoprotein C-II (APOC2) compared to MA and healthy controls (*p* = 0.03, *p* = 0.05, respectively) and down-regulation of apolipoprotein H (APOH) (*p* = 0.003, *p* = 0.0003, respectively) (Fig. 1). Notably, the standard deviation of APOH expression was considerably less in MAP patients. We also observed up-regulation of haptoglobin-related *protein* (HPTR) abundance in the MAP compared to MA (*p* = 0.04) (Fig. 1c).

### Diffusion tensor imaging

The same subjects all underwent DTI to retrieve information regarding brain white matter microstructure organization including fractional anisotropy (FA), mean diffusivity (MD), axial diffusivity (AD) and radial diffusivity (RD) across 48 ROI. FA reflects the orientation specificity of water diffusion in white matter where a decrease in FA can refer to white matter damage in relation to fiber tract coherence, fiber diameter, packing density and degree of myelination^51^. MD represents the degree of water diffusion regardless of fiber directionality where an increase in MD can indicate loss of white matter integrity relating to changes in intercellular space and compactness^52^. RD has been proposed as a marker of myelin content, and higher levels of diffusion perpendicular to the white matter tracts may be indicative of axon demyelination^53^. AD reflects diffusion parallel to the white matter tracks and has been proposed a marker of axonal integrity^51^.

**Figure 2:**
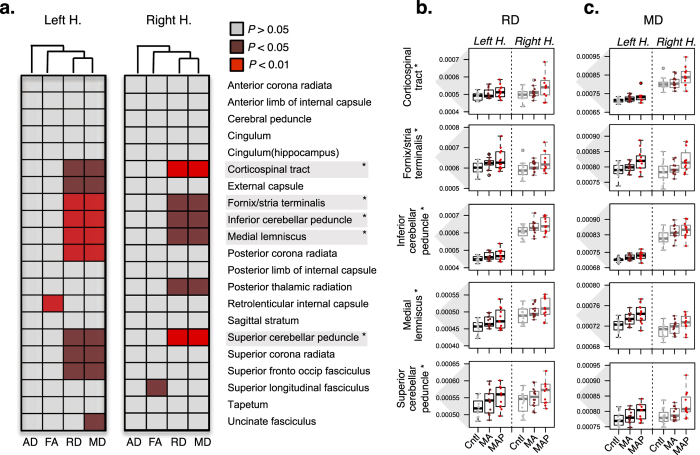
Diffusion tensor imaging (DTI) analysis. Mixed model analysis of variance (ANOVAs) compared MAP, MA and healthy controls (Cntl) for regions of interest (ROI), controlling for age, gender and polysubstance use variables (nicotine, alcohol, cannabis, mandrax). (**a**) ANOVA *P*-values were clustered (based on Pearson coefficient and average distance metric) accordingly to axial diffusivity (AD), fractional anisotropy (FA), radial diffusivity (RD) and mean diffusivity (MD) and a similar pattern is observed for both left and right hemispheres. Low ANOVA *P*-values are indicative of a difference across all groups and pairwise *post hoc* Tukey corrections were implemented to determine which group comparisons were significantly different. Following correction, the MAP group displayed bilateral high levels of (**b**) RD and (**c**) MD in several ROI (*), relative to MA and controls.

Following data pre-processing, we assessed whether regional mean DTI measurements differed between groups controlling for age, gender and polysubstance use variables (as described above). This analysis revealed group differences in MD and RD in several ROI and similar patterns of DTI alterations were detected in both hemispheres via hierarchical clustering of the resulting ANOVA *P*-values (Fig. 2a). After *post hoc* correction, the MAP group displayed bilateral high levels of MD and RD relative to MA and healthy controls in several ROIs including the corticospinal tract, fornix/stria terminalis, superior and inferior cerebellar peduncle and medial lemniscus (Fig. 2b,c). Similar high levels were observed in the fornix and the middle cerebellar peduncle (Supplementary Figure 2). Detailed results from all comparisons are displayed in Supplementary Table 3.

### Exploring relationships between serum proteins, clinical parameters and brain imaging

First, we investigated the relationships of serum protein abundance with clinical measures and DTI across all groups. While no proteins were associated with PANSS measures, several other proteins demonstrated nominally significant associations to EPQR-S scores (Supplementary Figure 3). When correlating protein abundance with DTI across all groups, APOH displayed the strongest extent of correlation to DTI measurements relative to all other proteins, and an overall positive correlation with FA and negative correlation with RD and MD for all ROIs was observed (Supplementary Figure 4a). After adjusting for multiple comparisons, three significant associations across all groups remained; APOH displayed significant negative associations with both RD and MD in the fornix (*r* = −0.50, *p* = 0.001; *r* = −0.53, *p* = 0.0001, respectively) as well as MD in the uncinate fasciculus (*r* = −0.47, *p* = 0.001) (Supplementary Figure 4d–f).

**Figure 3:**
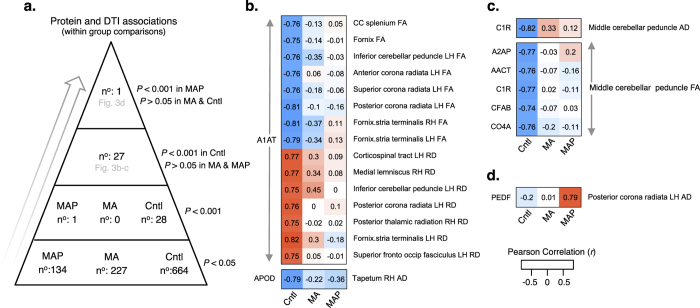
Exploratory associations between protein abundance and DTI within groups. (**a**) Stratification of associations according to a hierarchical *P*-value cut-off scheme. (**b**) A1AT displayed strong negative (with FA) and positive (with RD) associations to 15 ROI in healthy controls (Cntl), disrupted in MA and MAP groups. (**c**) Several proteins including A2AP, AACT, C1R, CFAB and CO4A negatively correlated to FA of the middle cerebellar peduncle in controls, with weak associations in MA and MAP groups. (**d**) PEDF positively correlated to AD in the left posterior corona radiata in MAP, not observed in MA or controls. Pearson correlations were Bonferroni corrected and *P*-values < 0.001 were designated significant. Corresponding *R*-values are displayed within each cell. Abbreviations; LH, left hemisphere; RH right hemisphere; AD, axial diffusivity; FA, fractional anisotropy; MD, mean diffusivity; RD, radial diffusivity.

Second, we examined associations between protein abundance and DTI studying groups separately (that is, within group comparisons). More nominally significant associations (*P* < 0.05) between protein abundance and DTI were identified in healthy controls (*n* = 664) compared to MA and MAP groups combined (*n* = 361) (two-tailed sign test; *p* = 0.001) (Fig. 3a). After adjusting for multiple comparisons, several significant associations were observed in healthy controls that were disrupted in MA and MAP groups (Fig. 3a–c). Of these associations, alpha-1 antitrypsin deficiency (A1AT) was associated with FA and RD in several ROIs in controls including the left corona radiate and fornix/stria terminalis. Additionally, numerous proteins including alpha 2-antiplamin (A2AP), alpha 1-antichymotrypsin (AACT), complement C1r (C1R), complement factor B (CFAB), complement component 4 A (CO4A) were strongly associated with AD and FA in the middle cerebellar peduncle in controls, but not in the MA or MAP groups. This exploratory analysis also identified a significant positive association between pigment epithelium-derived factor (PEDF) and AD in the left posterior corona radiata in MAP, but not in MA or control groups (Fig. 3d).

## Discussion

This study applied SRM-based proteomics in conjunction with DTI to assess the ability of these different measures to improve our mechanistic understanding of MAP and ultimately support development of better diagnostic and treatments. Our SRM-based quantitative proteomic assay monitors proteins with a greater level of sensitivity and specificity compared to conventional MS profiling, and measured proteins previously implicated in the etiology of major psychiatric disorders. We explored the hypothesis that serum proteins involved in processes of inflammation and lipid metabolism may be involved in the pathophysiology of MAP, and that these differences occur in parallel with white matter microstructural alterations. Protein analysis identified two candidate protein biomarkers, APOC2 and APOH, and DTI analysis indicated high levels in MD and RD delineating extensive loss of white matter integrity and axon demyelination in MAP. Integrating these data revealed several co-linear relationships between serum proteins and white matter microstructure in healthy controls, with disruption in MA and MAP groups in brain areas critical for memory and social emotional processing. These results suggest that changes in the serum occur in parallel to microstructural changes in the brain of MAP patients.

The majority of the protein analytes profiled in our SRM-based approach are involved in inflammation, lipid metabolism and immune system functions, consistent with the findings from previous studies^21^,^22^,^25^,^26^,^27^,^28^,^29^,^39^,^40^,^41^. The identified candidate biomarkers APOC2 and APOH displayed clear differences in MAP patients relative to MA and controls. APOC2 and APOH are from a diverse class of apolipoproteins, which play a well-established role in plasma lipoprotein metabolism and cholesterol homeostasis in the periphery^53^,^54^. They are also important for the bidirectional movement of cholesterol between the liver and peripheral tissues^53^,^54^. Alterations in APOC2 and APOH levels have been found in serum and plasma of patients diagnosed with SCZ^21^,^25^,^55^, bipolar disorder^26^ as well as several other disorders^29^,^56^,^57^. Notably, APOH was recently recognized as a part of a well validated 26 serum biomarker panel for classification of SCZ^21^. However, apolipoproteins are also implicated in pathways distinct from lipid metabolism. For example, inflammation markedly alters lipoprotein metabolism, and recent studies implicate lipoproteins as important mediators of the immune response and host defense mechanisms^58^. Increasing evidence indicates that APOC2 and APOH also fulfill a number of functions within the central nervous system and are critical for healthy brain function^53^,^54^,^59^, although their regulation and functional roles remain incompletely characterized.

With respect to potential mechanisms of apolipoproteins in MAP, one possibility is that activation of the sympathetic nervous system increases production of serum lipids and lipoproteins by altering lipid metabolic processes^60^. This hypothesis would support previous findings of increased norepinephrine in MAP patients^29^, with subsequent induction of lipolysis and release of free fatty acids into circulation, so providing substrate to resynthesize triglycerides and LDL production by the liver. This is also consistent with a growing body of work emphasizing the importance of psychosocial stress as a risk factor for psychosis. Alternatively, stress-associated elevations in serum lipoprotein productions may be attributable to concomitant hemoconcentration^61^. In support of this hypothesis, changes in serum lipid and lipoprotein levels reflect a filtration of fluid out of the intravascular space, by a passive increase in cholesterol, rather than an increase or decrease in the synthesis of these analytes per se^61^. A better mechanistic understanding of apolipoproteins in MAP is needed and may support therapeutic strategies that target the underlying lipid metabolism dysfunction, providing an alternative to treating the traditional neurotransmitter-related end-point of the disorder. Such therapeutic work may also consider measuring histones and methyltransferases to better understand epigenetic changes in MAP patients.

In parallel, DTI data implicated white matter microstructure changes in MAP patients relative to MA and healthy controls, despite few differences between MA and healthy controls. In MAP, high levels of MD and RD were observed in white matter tracts in the brain stem carrying information from the spinal cord to the cerebellum and thalamus, and between the cortex and cerebellum; and in association fibers carrying information between subcortical structures as part of the limbic system. While high levels of RD support the notion of axon demyelination in MAP, higher levels of MD support the concept of increases in intercellular space and in tissue water caused by inflammation or edema^50^. The cortical-thalamic-cerebellar-cortical circuit may play a pivotal role in the development of psychotic features in METH dependence, as it has also been implicated in the pathophysiology of SCZ (for review see refs 62,63). However, we have previously found evidence of more widespread white matter pathology in MAP^19^, supporting a dysconnectivity hypothesis in psychosis.

There have not been many efforts to establish the association between serum protein abundance and DTI to develop a multi-modal biosignature for MAP or METH dependence. In the present study, several strong associations were found between serum proteins and DTI in healthy controls, with weak to no correlation in MA or MAP groups (Fig. 3). Notably, A1AT showed correlations with FA and RD in several ROI in healthy controls, including the corona radiata, inferior cerebellar peduncle, and the fornix/stria terminalis. Numerous other proteins were also associated with AD and FA in the middle cerebellar peduncle in healthy controls, but not in MA or MAP, suggesting that co-linear relationships between serum proteins and DTI are more prevalent in healthy controls and are disrupted in the context of METH dependence. However, one strong positive correlation between PEDF and AD in the left posterior corona radiata of the MAP group was reported, but not identified in MA or healthy controls, indicating that this association may serve as an indicator of MAP. While some of these associations were drawn from regional mean DTI data which were not significantly different between groups, the corona radiata, cerebellar peduncle and fornix have previously been associated with impairments of memory^64^ and social emotion^65^,^66^ and implicated in several mental and substance use disorders^67^,^68^,^69^. These data demonstrate the possibility of identifying correlations between specific serum-based proteins and DTI to enhance our mechanistic understanding of METH dependence and to potentially improve diagnosis of MAP.

A particular strength of our study is the multiplex nature of SRM, which allows the highly accurate quantification of tens of proteins in a single experimental run allowing its use in both clinical trials and clinical pipelines for detection of diagnostic, prognostic and treatment-related biomarkers. Additionally, the inclusion of three distinct experimental groups (MAP, MA and healthy controls) matched for age and gender facilitated a well-controlled and balanced study. Although our sample size was small, previous studies conducted in serum, plasma and post-mortem brain tissue have also demonstrated the involvement of APOC2 and APOH across several psychiatric disorders^21^,^25^,^26^,^53^,^54^,^55^, further emphasizing the significance of our findings. Similarly, indices of diminished white matter integrity have been identified in critical brain regions, including the fornix, corona radiata and cerebellar peduncle, potentially implicated in the neuropathology of MAP.

Our exploratory study also has some limitations. It should be noted that antipsychotic drugs are known to induce metabolic side effects (*e.g.* insulin resistance, weight gain), and changes in levels of serum lipids have been correlated with clinical response to atypical antipsychotic treatment^70^. Although all MAP patients in this study were on haloperidol treatment at the time of testing, there is a dearth of studies linking altered APOC2 and APOH levels to antipsychotic treatment effects across human and rodent models. Notably, a recent study measuring the chronic effects of haloperidol treatment on the mouse hippocampi proteome identified hundreds of altered proteins^71^, however did not report differential patterns of APOC2 or APOH. Additionally, our previous report considered the contribution of haloperidol to blood-based RNA expression profiles across MAP, MA and control groups and found no evidence of treatment effecting differences in RNA levels^34^. Nevertheless, these findings reported here should be interpreted cautiously. Lastly, since this study leveraged a targeted panel of proteins previously implicated in SCZ^25^, changes in other proteins relevant to METH use (for review see ref. 32), such as SOD, GPx and brain-derived neurotrophic factor (BDNF), were not investigated, and these may also have significant roles in the pathophysiology of MAP.

In sum, this study represents an effort to unify these disparate data (often analyzed without integration), to characterize the physiological and neural underpinnings of MAP. Given the overlap between MAP and SCZ, our findings may shed light on the biological and neural mechanisms of psychosis in general. Future research using longitudinal designs, a range of different clinical populations including SCZ, and additional molecular mechanisms (e.g. epigenetics) is needed to further characterize mechanisms underlying the pathophysiology of MAP, and to determine the extent to which MAP provides a useful model for developing diagnostics and therapeutics for SCZ.

## Additional Information

**How to cite this article**: Breen, M. S. *et al*. Parallel changes in serum proteins and diffusion tensor imaging in methamphetamine-associated psychosis. *Sci. Rep.* **7**, 43777; doi: 10.1038/srep43777 (2017).

**Publisher's note:** Springer Nature remains neutral with regard to jurisdictional claims in published maps and institutional affiliations.

## Supplementary Material

Supplementary Figures

Supplementary Tables

## Figures and Tables

**Table 1:** Recorded demographic and clinical parameters for all participants (N = 42).

	Healthy Control (N = 16)	MA (N = 14)	MAP (N = 12)	ANOVA		*Post hoc Correction*
	Mean ± S.D.	Mean ± S.D.	Mean ± S.D.	X^2^ (*df* = *2*)	*P-value*	*Post hoc P-value*
Age	25.56 ± 5.99	22.71 ± 3.07	25 ± 7.39	1.565	0.457	ns
Gender: (*M/F*)	(14/2)	(10/4)	(9/3)	1.242	0.537	ns
Level of education (years)	12.13 ± 1.41	10.29 ± 1.98	9.75 ± 1.22	12.899	0.002	Control > MAP; Control > MA
EPQR-S Total	20.38 ± 5.08	19.93 ± 4.57	20.58 ± 3.92	0.761	0.683	ns
*EPQR-S Psychoticism*	2.56 ± 1.55	2.29 ± 1.86	2.83 ± 1.95	0.967	0.617	ns
*EPQR-S Extraversion*	9.94 ± 2.38	9.14 ± 2.25	7.17 ± 2.55	7.561	0.023	Control > MAP
*EPQR-S Neuroticism*	2.63 ± 2.36	4.86 ± 2.88	5.00 ± 2.92	6.355	0.032	MA > Control; MAP > Control
METH duration of use (years)	—	5.64 ± 2.31	6.46 ± 2.92	0.734	0.392	ns
Nicotine	7	9	12	9.583	0.008	MAP > Control
Cannabis	5	2	1	2.581	0.275	ns
Alcohol	6	7	1	5.125	0.077	ns
Mandrax	0	0	1	2.500	0.287	ns

Abbreviations: EPQRS, Eysenck Personality Questionnaire, short scale; METH, methamphetamine; MA, methamphetamine dependence without psychosis; MAP, methamphetamine-associated psychosis; ns, not significant. Shapiro-Wilk test was used to assess normality of variables and either a one-way analysis of variance (ANOVA) or Kruskal-Wallis ANOVA with post hoc Tukey correction were implemented accordingly.
