# Supplementary material for: Parallel changes in serum proteins and diffusion tensor imaging in methamphetamine-associated psychosis
Source: Sci Rep. 2017 Mar 2;7:43777. doi: 10.1038/srep43777 (PMC5333148; doi:10.1038/srep43777)
Supplement: Supplementary Figures [file srep43777-s1.pdf]

Supplementary Figures

Title: Parallel changes in serum proteins and diffusion tensor imaging in methamphetamine-associated psychosis

Authors: Michael S. Breen[1,2*]†, Anne Uhlmann [3]†, Sureyya Ozcan[4], Man Chan[4], Dalila Pinto[1,2,5,6], Sabine Bahn[3*], Dan J. Stein[3]

Affiliations:
[1]Division of Psychiatric Genomics, Department of Psychiatry, Icahn School of Medicine at Mount Sinai, New York, New York, USA.
[2]Seaver Autism Center for Research and Treatment, Icahn School of Medicine at Mount Sinai, New York, New York, USA.
[3]Department of Psychiatry and Mental Health, MRC Unit on Anxiety & Stress Disorders, University of Cape Town, South Africa.
[4]Institute of Biotechnology, University of Cambridge, United Kingdom.
[5]Institute for Genomics and Multiscale Biology, Department of Genetics and Genomic Sciences, Icahn School of Medicine at Mount Sinai, New York, New York, USA.
[6]Friedman Brain Institute, Icahn School of Medicine at Mount Sinai, New York, New York, USA.

†Both first authors equally contributed to this work.
*Corresponding authors.

Supplementary Figure 1. Quality control of protein expression across all three groups.
Supplementary Figure 2. Diffusion tensor imaging (DTI) analysis.
Supplementary Figure 3. Correlations between all proteins and EPQR-S measures of psychoticism, extraversion and neuroticism.
Supplementary Figure 4. Correlations between all proteins and DTI measurements.

For Supplementary Tables 1-3 please find separate supplementary table file which includes raw peptide level expressions across all participants (**Supplementary Table 1**), normalized protein level expression across all participants (**Supplementary Table 2**) and a detailed report of DTI comparisons between groups (**Supplementary Table 3**).

Supplementary Figure 1

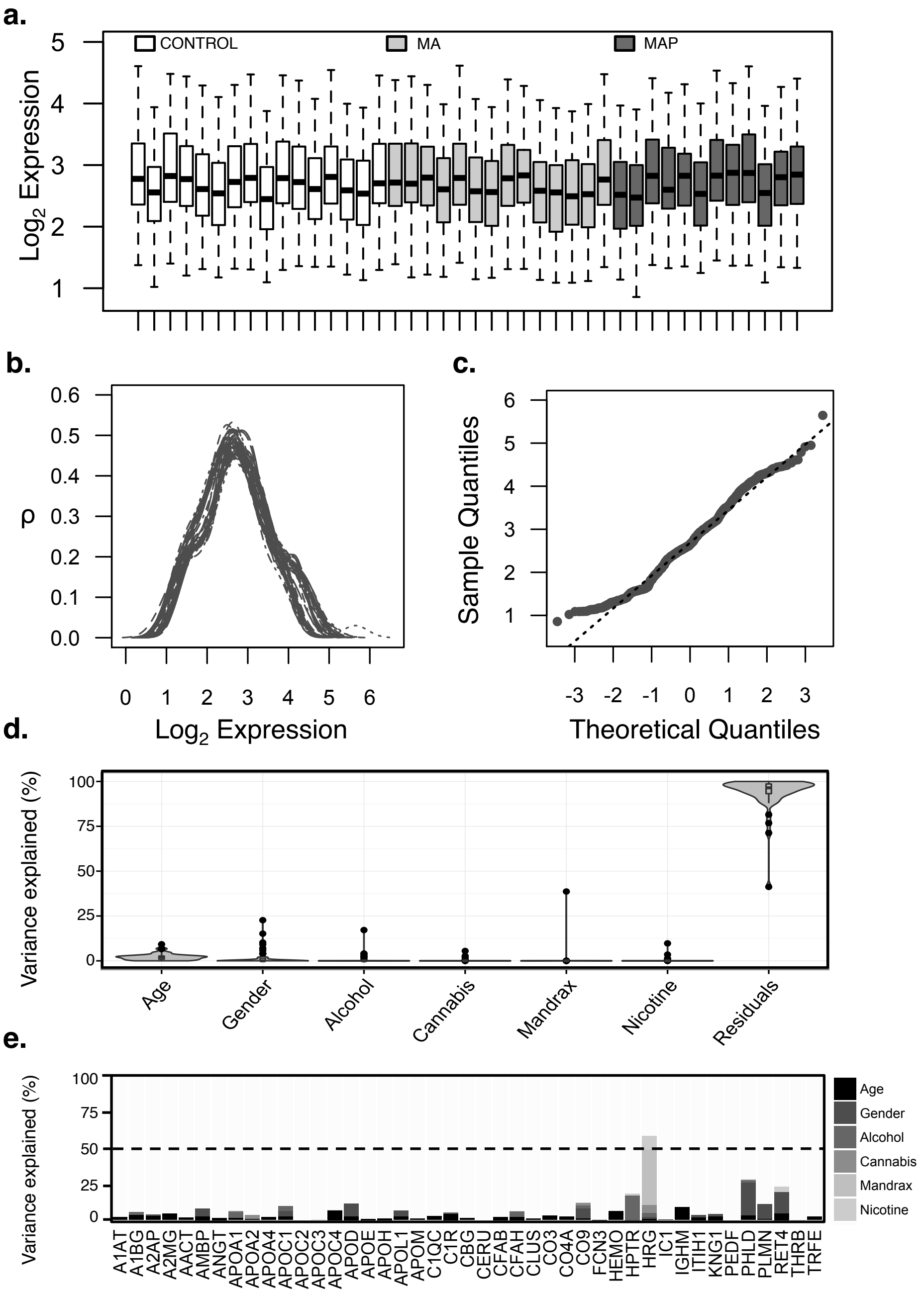

Supplementary Figure 1. Quality control of protein expression across all three groups. The distributions of normalized protein expression were inspected by (**a**) boxplots, (**b**) histograms of density distributions and (**c**) quantile-quantile plots. Age, gender and polysubstance use variables were investigated for their potential role in driving protein expression variation using the R package variancePartition. (**d**) The mean variance explained by all variables in relation to global protein expression profiles and (**e**) and their contribution per individual protein were calculated. Overall, the contribution of all variables were low and not likely to be confounding protein expression levels, although polysubstance use explained >50% variance of HRG protein levels.

Supplementary Figure 2

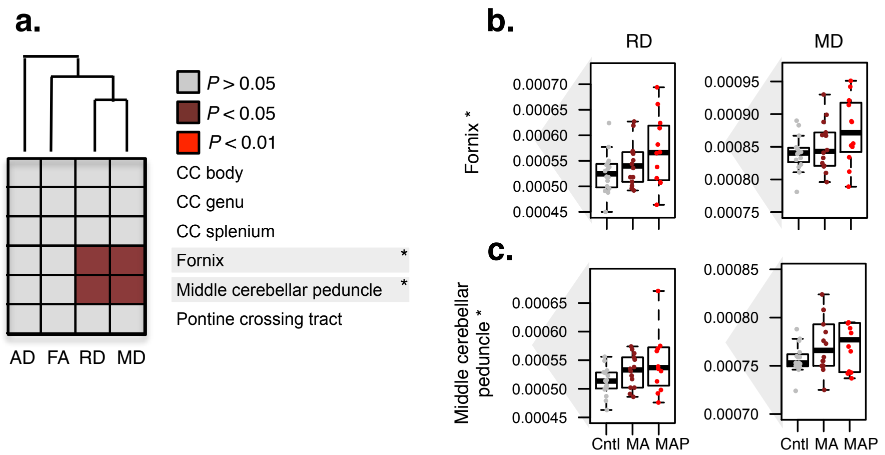

Supplementary Figure 2. Diffusion tensor imaging (DTI) analysis. Mixed model analysis of variance (ANOVAs) compared MAP, MA and healthy controls (Cntl) for regions of interest (ROI), controlling for age, gender and polysubstance use variables (nicotine, alcohol, cannabis, mandrax). (**a**) Resulting *P*-values were clustered (based on Pearson coefficient and average distance metric) accordingly to axial diffusivity (AD), fractional anisotropy (FA), radial diffusivity (RD) and mean diffusivity (MD). Low *P*-values are indicative of a difference across all groups and pairwise *poc hoc* Tukey corrections were implemented to determine which group comparisons were significantly different. Following correction the MAP group displayed increases in (**b**) RD and (**c**) MD in the fornix and middle cerebellar peduncle (*).

Supplementary Figure 3

	Psychoticism	Extraversion	Neuroticism
A1AT	-0.17 (0.3)	0.37 (0.02)	0.0036 (1)
A1BG	-0.27 (0.09)	-0.26 (0.1)	-0.24 (0.1)
A2AP	0.047 (0.8)	-0.083 (0.6)	0.34 (0.03)
A2MG	-0.13 (0.4)	-0.087 (0.6)	0.33 (0.03)
AACT	-0.037 (0.8)	-0.11 (0.5)	0.24 (0.1)
AMBP	-0.11 (0.5)	-0.12 (0.5)	0.27 (0.09)
ANGT	-0.079 (0.6)	-0.13 (0.4)	0.25 (0.1)
APOA1	-0.052 (0.7)	-0.18 (0.3)	0.35 (0.02)
APOA2	-0.36 (0.02)	-0.01 (0.9)	0.22 (0.2)
APOA4	-0.052 (0.7)	-0.046 (0.8)	0.33 (0.03)
APOC1	-0.078 (0.6)	-0.043 (0.8)	0.34 (0.03)
APOC2	-0.09 (0.6)	-0.26 (0.09)	0.093 (0.6)
APOC3	-0.06 (0.7)	-0.16 (0.3)	0.27 (0.08)
APOC4	0.12 (0.5)	-0.13 (0.4)	0.26 (0.09)
APOD	-0.042 (0.8)	0.0092 (1)	0.11 (0.5)
APOE	-0.13 (0.4)	-0.022 (0.9)	0.21 (0.2)
APOH	0.11 (0.5)	0.059 (0.7)	-0.099 (0.5)
APOL1	-0.055 (0.7)	-0.16 (0.3)	0.32 (0.04)
APOM	-0.08 (0.6)	-0.068 (0.7)	0.0022 (1)
C1QC	-0.041 (0.8)	-0.12 (0.5)	0.26 (0.1)
C1R	-0.022 (0.9)	-0.16 (0.3)	0.28 (0.07)
CBG	-0.057 (0.7)	-0.15 (0.3)	0.23 (0.2)
CERU	-0.091 (0.6)	-0.054 (0.7)	0.2 (0.2)
CFAB	-0.031 (0.8)	-0.14 (0.4)	0.26 (0.1)
CFAH	-0.1 (0.5)	-0.067 (0.7)	-0.21 (0.2)
CLUS	-0.053 (0.7)	-0.14 (0.4)	0.31 (0.05)
CO3	0.0035 (1)	-0.26 (0.1)	0.25 (0.1)
CO4A	0.011 (0.9)	-0.042 (0.8)	0.33 (0.03)
CO9	0.18 (0.3)	-0.11 (0.5)	0.047 (0.8)
FCN3	0.16 (0.3)	-0.32 (0.04)	0.059 (0.7)
HEMO	0.064 (0.7)	-0.11 (0.5)	0.071 (0.7)
HPTR	0.14 (0.4)	-0.14 (0.4)	0.14 (0.4)
HRG	-0.0057 (1)	0.27 (0.08)	0.18 (0.3)
IC1	0.033 (0.8)	-0.11 (0.5)	0.13 (0.4)
IGHM	0.14 (0.4)	-0.095 (0.5)	0.12 (0.4)
ITIH1	-0.079 (0.6)	-0.19 (0.2)	0.26 (0.09)
KNG1	-0.022 (0.9)	-0.18 (0.2)	0.29 (0.06)
PEDF	-0.074 (0.6)	-0.28 (0.08)	0.12 (0.4)
PHLD	-0.11 (0.5)	-0.085 (0.6)	0.26 (0.1)
PLMN	-0.13 (0.4)	0.15 (0.4)	-0.043 (0.8)
RET4	-0.18 (0.2)	-0.064 (0.7)	0.17 (0.3)
THRB	-0.044 (0.8)	-0.045 (0.8)	0.41 (0.007)
TRFE	-0.059 (0.7)	0.005 (1)	0.22 (0.2)

Supplementary Figure 3. Correlations between all proteins and EPQR-S measures of psychoticism, extraversion and neuroticism. Each box contains a pairwise correlation between protein and EPQR-S measure with the top number indicating strength of correlation (*r*) and the bottom number indicating significance of corresponding correlation (*P*-value). Red indicates positive correlation and blue indicates negative correlation. Spearman correlations were applied and displayed *P*-values are not corrected for multiple comparisons. No significant correlations were observed between protein and PANSS measurements.

Supplementary Figure 4

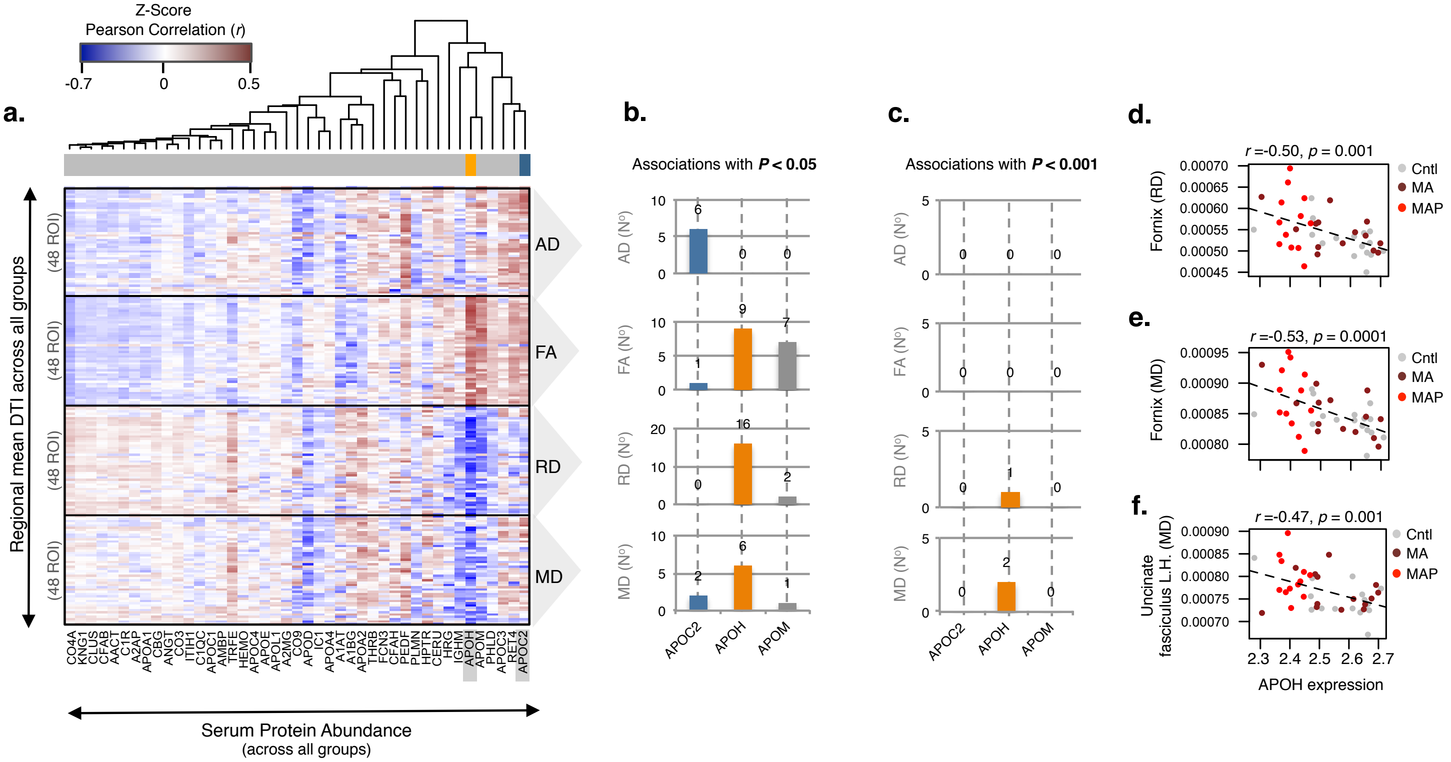

Supplementary Figure 4. Correlations between all proteins and DTI measurements. (**a**) Pearson correlation coefficients were computed between protein abundance and DTI and clustered (based on Pearson coefficient and average distance metric) accordingly to proteins with similar strengths of association to DTI parameters. Red indicates positive correlation, blue indicates negative correlation. Protein APOC2 is colored orange and APOH is colored dark blue. Total number of significant associations were counted surviving *P*-values (**b**) <0.05 and (**c**) <0.001 for proteins of interest. Of associations passing $P < 0.001$, APOH was negatively correlated with (**d**) radial diffusivity (RD) in the fornix, (**e**) mean diffusivity (MD) in the fornix and (**f**) MD in the left (L.H.) uncinate fasciculus. Color code: grey, controls; brown MA; red, MAP.
